# Supplementary material for: Novel Pilot-Scale Photocatalytic Nanofiltration Reactor for Agricultural Wastewater Treatment
Source: Membranes (Basel). 2023 Feb 6;13(2):202. doi: 10.3390/membranes13020202 (PMC9966609; doi:10.3390/membranes13020202)
Supplement: Supplementary file 1 [file membranes-13-00202-s001.zip › membranes-2165273-supplementary.pdf]

membranes

Supplementary Materials

Novel Pilot-Scale Photocatalytic Nanofiltration Reactor for Agricultural Wastewater Treatment

George V. Theodorakopoulos [1,2,*], Michalis K. Arfanis [1], José Antonio Sánchez Pérez [3,4], Ana Agüera [3], Flor Ximena Cadena Aponte [3], Emilia Markellou [5], George Em. Romanos [1,*] and Polycarpos Falaras [1,*]

1 Institute of Nanoscience and Nanotechnology, National Center of Scientific Research "Demokritos", Agia Paraskevi 15310, Athens, Greece
2 Inorganic and Analytical Chemistry Laboratory, School of Chemical Engineering, National Technical University of Athens, Zografou Campus, 9 Iroon Polytechneiou str., Zografou 15772, Athens, Greece
3 Solar Energy Research Centre (CIESOL), Joint Centre University of Almería-CIEMAT, Carretera de Sacramento s/n, E-04120, Almería, Spain
4 Chemical Engineering Department, University of Almería, Carretera de Sacramento s/n, E-04120, Almería, Spain
5 Laboratory of Mycology, Scientific Directorate of Phytopathology, Benaki Phytopathological Institute, 8 St. Delta str., Kifissia 14561, Athens, Greece
* Correspondence: g.theodorakopoulos@inn.demokritos.gr (G.V.T.); g.romanos@inn.demokritos.gr (G.Em.R.); p.falaras@inn.demokritos.gr (P.F.); Tel.: +30-210-650-3977 (G.V.T.); +30-210-650-3972 (G.Em.R.); +30-210-650-3644 (P.F.)

S1. Analytical Method

The concentration of the pesticides was measured by UHPLC-DAD device (Series 1200 coupled to diode-array detector, Agilent Technologies, Foster City, CA, USA) using a linear gradient from 100% phase A to 100% phase B in 6.1 min. A HPLC reversed-phase XDB-C18 analytical column (Zorbax Eclipse XDB-C18, 1.8 μm, 4.6 × 50 mm, Agilent Technologies, Foster City, CA, USA) was used. The mobile phase used was a mixture of solvents A (ultrapure water acidified with 0.1% formic acid) and B (acetonitrile). The flow rate was 1 mL/min and the injection volume was 100 μL. The UV-signal for each compound was recorded at the wavelength of maximum absorption: 300 and 248 for thiabendazole and acetamiprid, respectively. Before UPLC analysis, 9 mL of sample were filtered (0.22 μm) and the filter was then washed with 1 mL of acetonitrile (ACN) for a final volume of 10 mL (9:1 sample:ACN).

S2. Photoluminescence (PL) experiments

Fluorescence spectroscopy measurements were performed employing a Jasco FP-8300 spectrofluorometer (Jasco, Tsukuba, Japan) at room temperature. The obtained results summarized in the Figure below (Figure S1) show the corresponding PL spectra for the three samples (sol, P25 and sol/P25). Following the literature [1], a strong emission intensity is usually associated with high electron-hole recombination probability. However, our results show that the sol sample presents the lower PL intensity. It must be taken into account that even if we have used the same quantities for the catalysts, their dispersions in the corresponding samples were not equivalent. Indeed, the materials differ in terms of physicochemical properties that might affect the PL emission (e.g., scattering effects). It is then clear that for sol sample the obtained PL data do not fully support the fast complexation of electron-hole pairs of sol compared to P25 and sol/P25. To better understand its moderate catalytic activity, it should be taken into consideration that the sol sample is crystallized only in the anatase phase, so it has different electronic states, and might have different PL behavior and photogenerated radicals' formation mechanism. Moreover, the surface chemistry of sol sample might differ from the P25-based materials, so another plausible explanation might be the pH zero charge point and the ability to adsorb the target molecules.

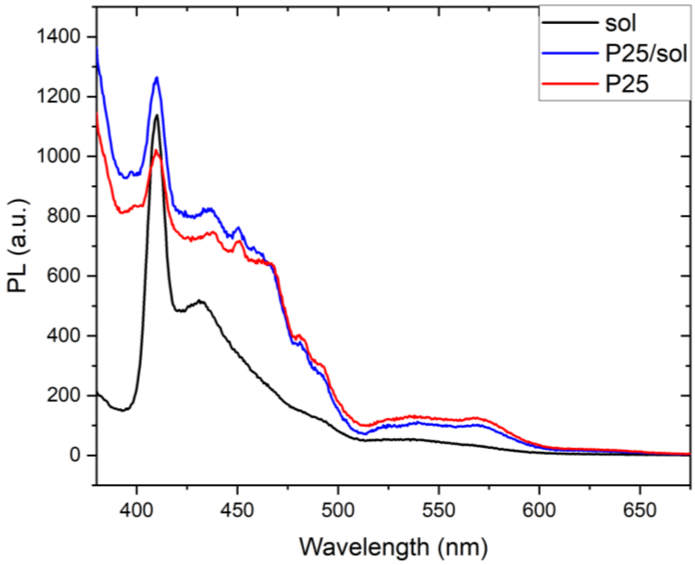

Figure S1. PL emission spectra of three samples (sol, P25 and sol/P25).

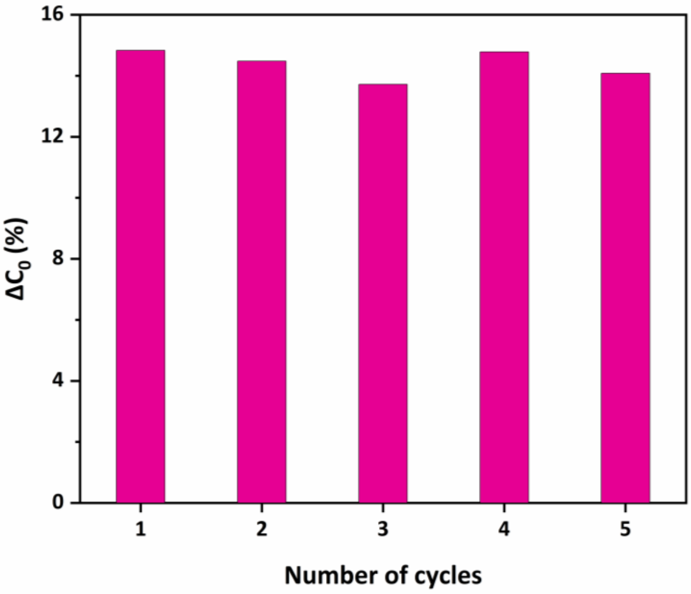

Figure S2. Final RhB rejection (%) due to photocatalytic degradation by PHFs under UV-A illumination for 90 min, after five successive cycles (C_0 = 5 ppm, pH = 6, 25 °C).

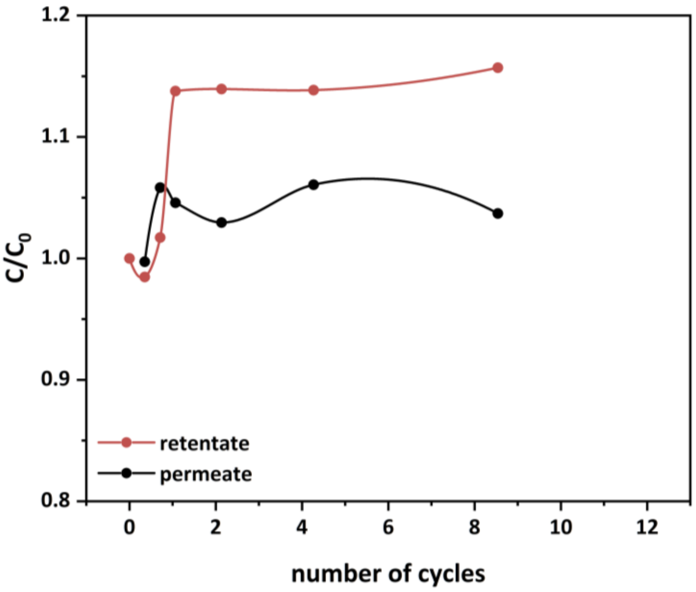

Figure S3. Micropollutant's rejection evolution in permeate and retentate effluents during the number of operation cycles of the PNFR unit without complete regeneration of the unit.

Reference

1. I. Papailias, N. Todorova, T. Giannakopoulou, N. Ioannidis, P. Dallas, D. Dimotikali, C. Trapalis, Novel torus shaped g-C_3N_4 photocatalysts, *Appl. Cat. B: Env.*, **2020**, *268*, 118733.
